# Hospital treatment costs and length of stay associated with hypertension and multimorbidity after hemorrhagic stroke

**DOI:** 10.1186/s12883-017-0930-2

**Published:** 2017-08-10

**Authors:** Adrian V. Specogna, Tanvir C. Turin, Scott B. Patten, Michael D. Hill

**Affiliations:** 1 Department of Health Professions, College of Health and Public Affairs, University of Central Florida, 12805 Pegasus Drive, Orlando, FL 32816 USA; 2 Department of Family Medicine, Cumming School of Medicine, University of Calgary, 3330 Hospital Drive NW, Calgary, AB T2N 4N1 Canada; 3 Department of Community Health Sciences, Cumming School of Medicine, University of Calgary, 3280 Hospital Drive NW, Calgary, AB T2N 4Z6 Canada; 4 Department of Clinical Neurosciences, Cumming School of Medicine, Foothills Hospital, Room 1242A, 1403 29th Street NW, Calgary, AB T2N 2T9 Canada

**Keywords:** Intracerebral hemorrhage, Cost of care, Comorbidity, Hypertension, Critical care, Economics, Epidemiology

## Abstract

**Background:**

Previous studies have identified various treatment and patient characteristics that may be associated with higher hospital cost after spontaneous intracerebral hemorrhage (ICH); a devastating type of stroke. Patient morbidity is perhaps the least understood of these cost-driving factors. We describe how hypertension and other patient morbidities affect length of stay, and hospital treatment costs after ICH using primary and simulated data. We also describe the relationship between cost and length of stay within these patients.

**Methods:**

We used a cohort design; evaluating 987 consecutive ICH patients across one decade in a Canadian center. Economic, treatment, and patient data were obtained from clinical and administrative sources. Multimorbidity was defined as the presence of one or more diagnoses at hospital admission in addition to a primary diagnosis of ICH.

**Results:**

Hypertension was the most frequent (67%) morbidity within these patients, as well as the strongest predictor of longer stay (adjusted RR for >7 days: 1.31, 95% CI: 1.07-1.60), and was significantly associated with higher cost per visit when accounting for other morbidities (adjusted cost increase for hypertension $8123.51, 95% CI: $4088.47 to $12,856.72 USD). A Monte Carlo simulation drawing one million samples of patients estimated for a generation (100 years) assuming 0.94% population growth per year, and a hospitalization rate of 12 per 100,000 inhabitants, supported these findings (*p* = 0.516 for the difference in unadjusted cost: simulated vs primary). Using a restricted cubic spline, we observed that the rate of change in overall cost for all patients was greatest for the first 3 weeks (*p* < 0.001) compared to subsequent weeks.

**Conclusion:**

Patient multimorbidity, specifically hypertension, is a strong predictor of longer stay and cost after ICH. The non-linear relationship between cost and time should also be considered when forecasting healthcare spending in these patients.

## Background

Spontaneous intracerebral hemorrhage (ICH) is a devastating and costly condition with high mortality and morbidity. Previous studies have attempted to identify various patient or treatment characteristics that may be associated with higher hospital costs to assist in economic planning [1–3]. Patient comorbidity has been cited as an important cost-driving factor [2], however the details of its association with hospital resource use remains unclear.

Hypertension is arguably the strongest risk factor for the development of ICH and thus the most common secondary disease patients present with in the emergency department [4]. How specific comorbid or ICH-associated conditions, such as hypertension affect length of stay in hospital, or cost of specific services has yet to be reported and thus there little understanding of the extent of care for these patients, and thus little understanding of how new promising treatment strategies [5] for such conditions could affect hospital resource use once implemented. Further, identifying differences in how specific morbidities are associated with specific costs could reveal differences in care amongst some patients. This cost information could provide insight into ICH care efficiency; both within and beyond emergency departments. The primary purpose of this study was to describe how patient morbidities, specifically hypertension, affect length of stay, and treatment cost after ICH. We also describe as a secondary objective, the relationship between cost and length of stay, and explore the potential relationship between stroke severity, clinical outcomes, and cost within these patients.

## Methods

We used a cohort study design to evaluate the economic cost of ICH hospital care within one Canadian hospital between 1999 and 2008. This center is one of two dedicated stroke centers in the province of Alberta, and is located in a city with a population of approximately one million people overall. The cost of care was estimated retrospectively from the time of admission to the time of discharge using administrative data provided by the province and included both direct and indirect expenditures combined.

In order to ensure our economic estimates were precise and selection bias was minimized, we sought to obtain all financial data on all ICH cases treated during the decade. This center is one of several centers nationally that captures micro-costing data on all hospital admissions; thus detailed cost data were available from administrative sources. Capturing all economic data is particularly important for costing studies of ICH since the variability in ICH economic data is high [3], and if the number of ICH cases observed is low, cost estimates may be imprecise and uninformative. Thus, all adult (> = 18 years) hospitalized patients with a Most Responsible Diagnosis of ICH (ICD-9-CM and ICD-10-CA: 431, I61.0-I61.6, I61.8, and I61.9) were eligible for inclusion. Patients were excluded only if the cost of treatment could not be estimated or was not reported to the provincial government.

The provincial government follows national guidelines [6] for the collection, coding and quality assurance of patient demographic and comorbidity data, and provides the ability to link such data to information provided by different departments within its organization. In this study, the costs associated with hospital stays were provided by the provincial health-costing department and linked to an electronic patient record. Financial data were inflated to the year 2015 using the Consumer Price Index [7], to adjust for overall economic inflation.

Ordinary binomial regression was used to investigate the association between specific diseases recorded at hospital admission (yes vs. no), and length of stay (<= 7 days vs. >7 days), and calendar year. Ordinary linear regression was used to investigate the association between these diseases and inflation-adjusted cost overall, and within specific costed categories. For our primary morbidity of interest, hypertension, we explored the potential confounding effect of stroke severity, in-hospital mortality, and disability on its relationship with cost and length of stay using ordinary linear regression and stratified analysis. We also performed a Monte Carlo sensitivity analysis to investigate the association between cost and hypertension; which was informed using current ICH summary data from different centers.

All analyses were conducted using Stata statistical software [8] assuming alpha was equal to 0.05. For the purpose of this study, multimorbidity was defined as the presence of one or more diseases, which patients experienced, in addition to the index ICH [9]. Decisions for morbidity classification and stratification were informed from previous investigations [2, 10, 11] (Table 1). As expected, all cost data were right skewed thus they were log (ln) transformed for analysis. The significance of cost increases and decreases were determined using robust standard errors. Log costs were reported using smear retransformation [12] to provide realistic cost values from linear regression and reported per discharge in United States Dollars (USD) at December 31, 2015 to allow for international comparison. All those individuals who reported costs and measured clinical variables were blinded to the study objectives. We obtained approval and a waiver of written consent from the University of Calgary’s Conjoint Health Research Ethics Board to conduct this study.

**Table 1:** International Classification of Diseases codes (ICD-9-CM and ICD-10-CA) used to classify patient morbidity

Morbidity Category	ICD-9-CM and ICD-10-CA Codes
Hypertension	401.x, 402.x, 403.x, 404.x, 405.x, I10.x, I11.x, I12.x, I13.x, I15.x
Secondary Cerebrovascular Disease ^a^	362.34, 430.x–438.x, G45.x, G46.x, H34.0, I60.x–I69.x
Diabetes	250.0–250.3, 250.4–250.7, 250.8, 250.9, E10.0–E10.9, E11.0–E11.9, E12.0- E12.9, E13.0–E13.9, E14.0-E14.9
Cardiac Disorders	398.91, 402.01, 402.11, 402.91, 404.01, 404.03, 404.11, 404.13, 404.91, 404.93, 410.x, 412.x, 425.4–425.9, 428.x, I09.9, I11.0, I13.0, I13.2, I25.5, I21.x, I22.x, I25.2, I42.0, I42.5–I42.9, I43.x, I50.x, P29.0
Chronic Pulmonary Disease	416.8, 416.9, 490.x–505.x, 506.4, 508.1, 508.8, I27.8, I27.9, J40.x–J47.x, J60.x–J67.x, J68.4, J70.1, J70.3
Malignancy or Tumour	140.x–172.x, 174.x–195.8, 196.x–199.x, 200.x–208.x, 238.6, C00.x–C26.x, C30.x–C34.x, C37.x–C41.x, C43.x, C45.x–C58.x, C60.x–C76.x, C77.x–C80.x, C81.x–C85.x, C88.x, C90.x–C97.x
Dementia	290.x, 294.1, 331.2, F00.x–F03.x, F05.1, G30.x, G31.1
Mood Disorders	296.x, 311.x, F30.x, F31.x, F32.x, F33.x, F34.x, F38.x, F39.x
Renal Disease	403.01, 403.11, 403.91, 404.02, 404.03, 404.12, 404.13, 404.92, 404.93, 582.x, 583.0–583.7, 585.x, 586.x, 588.0, I12.0, I13.1, N03.2–N03.7, N05.2–N05.7, N18.x, N19.x, N25.0, V42.0, V45.1, V56.x, Z49.0–Z49.2, Z94.0, Z99.2
Peripheral Vascular Disease	093.0, 437.3, 440.x, 441.x, 443.1–443.9, 447.1, 557.1, 557.9, I70.x, I71.x, I73.1, I73.8, I73.9, I77.1, I79.0, I79.2, K55.1, K55.8, K55.9, V43.4, Z95.8, Z95.9
Liver Disease	070.22, 070.23, 070.32, 070.33, 070.44, 070.54, 070.6, 070.9, 456.0–456.2, 570.x, 571.x, 572.2–572.8, 573.3, 573.4, 573.8, 573.9, B18.x, I85.0, I85.9, I86.4, I98.2, K70.0–K70.4, K70.9, K71.1, K72.1, K72.9, K76.5, K76.6, K76.7, K71.3–K71.5, K71.7, K73.x, K74.x, K76.0, K76.2–K76.4, K76.8, K76.9, V42.7, Z94.4
Rheumatic Disease	446.5, 710.0–710.4, 714.0–714.2, 714.8, 725.x, M05.x, M06.x, M31.5, M32.x–M34.x, M35.1, M35.3, M36.0
Peptic Ulcer Disease	531.x–534.x, K25.x–K28.x

^a^Patients with ICH in two or more locations were classified as having primary ICH with multimorbid secondary cerebrovascular disease

## Results

One thousand and two ICH patients were treated at the center during the decade. Cost data were not available in 15 patients; thus they were excluded. The characteristics of the 987 patients included are described in Table 2. The median total inflation-adjusted cost of care per stay (discharge) was $10,202.73 ($351.76 [min] to $256,867.22 [max]). Two percent of patients had survived an ICH previous to their current admission, although recurrent ICH was not associated with overall cost (*p* = 0.109).

**Table 2:** Sample characteristics

Patient Characteristic	Overall (*n* = 987)
Sex (% Males)	55
Age (Years)	72 (20-99)
Length of Stay in Hospital (Days)	8 (1-190)
Died in Hospital (%)	28^*^
Had Surgery (%)	18^*^
Accessed Diagnostic Services (%)	
Diagnostic Investigations	88
Laboratory Services	97
Overall	99
Accessed Acute Care Services (%)	
Ambulatory Care	3^**^
Clinical Nutrition	41^*^
Nursing	100
Pharmacy	85^**^
Overall	100
Accessed Rehabilitation Services (%)	
Audiology and Speech Therapy	38
Occupational Therapy	62^**^
Physiotherapy	70
Recreation Therapy	2
Respiratory Therapy	42^*^
Social Work	45
Overall	88
Morbidities at Admission (%)	
Hypertension	67
Secondary Cerebrovascular Disease	23
Diabetes	15
Cardiac Disorders	10^*^
Chronic Pulmonary Disease	6^*^
Malignancy or Tumour	5
Dementia	5
Mood Disorders	3^*^
Renal Disease	3
Peripheral Vascular Disease	2^*^
Liver Disease	1
Rheumatic Disease	1
Peptic Ulcer Disease	1^*^
Overall	81

Data are reported as median (min to max) unless otherwise noted. Percentages are rounded to the nearest whole number. Diseases were classified at hospital admission. Some patients may be classified in multiple categories. Overall morbidity indicates the percentage of patients who had at least one additional diagnosis irrespective of their primary diagnosis of ICH. Of the 15 patients who did not access diagnostic services; 8 died within the first 2 days, 3 were referred from other centers, 1 died with dementia within 5 days, 1 died within 6 days with no multimorbidity, 1 was discharged alive with a malignancy or tumour, and 1 was discharged alive with no multimorbidity

*Significant (*p* < 0.05) decrease over a decade

**Significant (*p* < 0.05) increase over a decade

### Morbidity, length of stay, and cost

The majority (81%) of patients had at least one additional diagnosis irrespective of their primary diagnosis of ICH at hospital admission (median: 1, 0 [min] to 6 [max]). Sixty-seven percent of all patients were hypertensive; which was the most frequent multimorbidity (Table 2). We felt it was reasonable to assume age could be associated with cost, and thus considered age as a confounder in all of our cost analyses. In contrast, sex was not associated with cost in our cohort. When adjusting for age, hypertension was significantly associated with higher cost (dollar increase in total cost per discharge: $10,324.56, 95% CI: $5586.55 to $15,862.53, Table 3). ICH patients with at least one additional disease were more likely to stay in hospital longer compared to those without any multimorbidity (RR for longer stay: 1.69, 95% CI: 1.36-2.10) and these patients were significantly more costly to treat at all stages of care (Table 3). When making the assumption that each morbidity contributed equally to cost, overall cost increased significantly for each added morbidity (age-adjusted cost increase per added morbidity: $4958.36, 95% CI: $3144.97 to $6907.00).

**Table 3:** Cost of patient morbidities measured at admission

Multimorbidity	RR for In-Hospital Mortality (95% CI)	Days Spent In Hospital	RR for Longer Stay (95% CI)	Dollar Change in Cost Diagnostic Services (95% CI)	Dollar Change in Cost Acute Care Services (95% CI)	Dollar Change in Cost Rehabilitation Services (95% CI)	Dollar Change in Cost Overall (95% CI)	*P*-Value for Dollar Change in Cost Overall
Hypertension	0.86 (0.69-1.06)	9 (4-20)	1.32^*^ (1.14-1.53)	$1846.01^*^ ($321.66 to $3817.97)	$6567.21^*^ ($3432.52 to $10,296.64)	$3426.76^*^ ($1621.76 to $6667.09)	$10,324.56^*^ ($5586.55 to $15,862.53)	*p* < 0.001
Secondary Cerebrovascular Disease	1.41^*^ (1.14-1.75)	9 (4-21)	1.08 (0.95-1.24)	$2559.95^*^ ($1078.05 to $4401.97)	$4268.28^*^ ($1015.40 to $8195.50)	$1020.98^*^ ($187.84 to $2477.67)	$8675.26^*^ ($3557.60 to $14,724.72)	*p* < 0.001
Diabetes	0.86 (0.64-1.17)	10 (5-26)	1.18^*^ (1.02 to 1.36)	$1127.79 (−$662.19 to $3586.67)	$5658.97^*^ ($1220.92 to $11,308.39)	$2826.20^*^ ($1309.58 to $5341.17)	$7581.65^*^ ($7835.58 to $15,357.99)	*p* = 0.016
Cardiac Disorders	1.40^*^ (1.06-1.85)	12 (4-27)	1.14 (0.96-1.35)	$3754.75^*^ ($1028.21 to $7613.74)	$4030.50 (−$1004.88 to $10,796.07)	$1004.67 (−$67.81 to $3191.30)	$8259.74^*^ ($531.06 to $18,195.80)	*p* = 0.034
Chronic Pulmonary Disease	1.39 (0.99-1.96)	9 (4-20)	1.09 (0.87-1.36)	$3005.11^*^ ($1183.36 to $5317.47)	-$2736.45 ($7296.18 to $4283.75)	$2143.12^*^ ($573.89 to $5172.46)	$1069.32 (−$6346.84 to $11,195.36)	*p* = 0.804
Malignancy or Tumour	1.32 (0.87-1.99)	10 (3-43)	1.08 (0.83-1.40)	-$1267.34 (−$3801.80 to $4622.35)	$3830.40 (−$3029.04 to $14,479.19)	-$97.25 (−$831.66 to $2529.73)	$4521.52 (−$5750.61 to $19,924.11)	*p* = 0.443
Dementia	0.97 (0.64-1.49)	14 (5-26)	1.19 (0.97-1.46)	$78.64 (−$2260.05 to $4060.67)	$3840.99 (−$2810.53 to $13,882.24)	$1249.18 (−$20.97 to $3929.90)	$5346.22 (−$3998.06 to $18,509.50)	*p* = 0.299
Mood Disorders	1.07 (0.59-1.94)	17 (6-44)	1.37^*^ (1.06-1.75)	$3090.06^*^ ($719.04 to $6364.49)	$10,112.11 (−$836.62 to $29,273.87)	$3440.20^*^ ($344.54 to $13,369.57)	$16,818.57^*^ ($1569.25 to $40,450.14)	*p* = 0.026
Renal Disease	1.48 (0.96-2.28)	9 (5-42)	1.14 (0.84-1.54)	$2610.50 (−$177.15 to $6873.98)	$8763.22 (−$1039.77 to $25,357.48)	$1520.56 (−$353.38 to $8403.06)	$14,969.86^*^ ($69.12 to $38,456.89)	*p* = 0.049
Peripheral Vascular Disease	1.16 (0.66-2.06)	10 (7-15)	1.24 (0.93-1.64)	$6391.68^*^ ($3331.82 to $10,504.63)	$298.49 (−$5061.45 to $8352.73)	-$544.78 (−$988.97 to $1578.40)	$2218.22 (−$6135.35 to $13,973.98)	*p* = 0.646
Liver Disease	1.55 (0.76-3.16)	11 (8-18)	1.64^*^ (1.21-2.23)	$3181.54 (−$128.61 to $8541.41)	$10,737.56 (−$1195.32 to $32,732.61)	$4186.12 (−$319.55 to $37,111.26)	$13,254.18 (−$4186.59 to $44,542.85)	*p* = 0.169
Rheumatic Disease	1.76 (0.78-3.95)	17 (5-45)	1.24 (0.71-2.18)	-$406.54 (−$4072.03 to $12,134.32)	$9393.44 (−$3464.65 to $35,755.59)	$5273.28^*^ ($1630.79 to $13,757.05)	$10,184.51 (−$9610.37 to $53,007.91)	*p* = 0.410
Peptic Ulcer Disease	1.01 (0.32-3.24)	16 (9-39)	1.62^*^ (1.18-2.24)	$9010.32^*^ ($6377.15 to $12,224.36)	$14,473.14 (−$1563.28 to $48,733.45)	$988.84 (−$1033.07 to $58,779.38)	$21,867.99 (−$544.05 to $63,578.35)	*p* = 0.058
Overall	1.12 (0.85-1.49)	9 (4-21)	1.69^*^ (1.36-2.10)	$3701.07^*^ ($1500.94 to $6854.65)	$9777.19^*^ ($6156.57 to $14,204.96)	$4949.90^*^ ($2080.47 to $11,239.82)	$16,388.39^*^ ($10,772.65 to $16,388.39)	*p* < 0.001

Days spent in hospital are reported as median (interquartile range). Longer stay is defined as <=7 days vs. >7 days. The relative risk (RR) for longer stay and in-hospital mortality is defined as the risk for those with the morbidity compared to those without. Cost estimates are for both direct and indirect expenditures combined. All estimates were adjusted for patient age as a continuous variable. CI denotes confidence interval. Confidence intervals which encompass zero suggest no evidence of a dollar change in cost

*Significant (*p* < 0.05)

After adjusting for age and all other comorbid diagnoses listed in Table 3, to address the issue that some ICH patients may have multiple secondary diseases simultaneously, hypertension remained the strongest predictor of longer stay (age and additional morbidity adjusted RR: 1.31, 95% CI: 1.07-1.60) and, along with secondary cerebrovascular disease, remained significantly associated with higher cost overall (adjusted dollar increase for hypertension: $8123.51, 95% CI: $4088.47 to $12,856.72, and for secondary cerebrovascular disease: $6637.13, 95% CI: $2541.79 to $11,502.75).

### Cost and length of stay

**Fig. 1:**
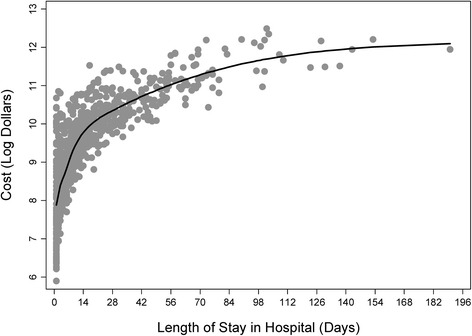
Total treatment cost vs. length of stay in hospital. Scatter plot of log (ln) total inflation-adjusted cost of hospital care and days spent in hospital after ICH. The plot shows a non-linear relationship between log cost and length of stay with cost variability being the highest within the first few days after ICH. The shaded area represents the raw data and the solid line represents the average through the data using an unadjusted restricted cubic spline function

The relationship between overall cost and time spent in hospital is described in Fig. 1. As illustrated, the relationship between cost and time was not linear, thus we estimated the average change in cost over time using a restricted cubic spline function (model R^2^: 0.73, Fig. 1). For ease of interpretation, we also estimated a linear spline function using weekly intervals; making the assumption that the association between cost and time was linear within each respective interval. According to this model, the rate of change in cost per day was significant for the first 3 weeks (*p* < 0.001 for linear slope for days 0-7, 8-14, and 15-21), but not significant compared to the previous interval for the rest of the follow-up period; suggesting that although cost continues to increase over time, the rate of this increase is not significant beyond 3 weeks after ICH. The median cost of care during the first week, first 2 weeks, and first 3 weeks were $4685.52 (interquartile range: $2761.84 to $7196.43), $6435.37 (interquartile range: $3592.59 to $11,248.09), and $8867.64 (interquartile range: $3867.85 to $13,612.64) respectively. Table 4 illustrates the median and average total cost for other commonly reported follow-up intervals.

**Table 4:** Cost of hospital treatment during the first day, first week, first month, and first year after ICH

Length of Stay	No. of Patients	Median Cost	Mean Cost
0-1 Day after ICH	90	$1909.45 ($1085.58 to $5456.15)	$5457.47 ± $8144.59 ($351.76 to $42,090.59)
0-30 Days after ICH	850	$8397.25 ($4159.22 to $16,829.43)	$12,355.65 ± $12,168.34 ($351.76 to $98,207.53)
0-365 Days after ICH	987	$10,202.73 ($4821.50 to $22,905.50)	$20,165.14 ± $28,433.37 ($351.76 to $256,867.19)

Data are unadjusted and presented as median (interquartile range) and mean ± standard deviation (min to max)

### Exploratory analysis of stroke severity, death, and disability

We ran additional regression analyses on a consecutive series of patients who had detailed clinical information (*n* = 148) obtained during routine care to investigate the potential affect of stroke severity, in-hospital mortality, and disability on the association between hypertension and cost, and length of stay; to determine whether these characteristics may be acting as confounders. The mean log total cost of this subgroup was not different than the entire sample of patients (*p* = 0.298) and hypertension, the most common multimorbidity, was significantly associated with length of stay in the subgroup (*p* = 0.032), and significantly associated with overall cost in the subgroup (*p* = 0.021), as it was in the entire cohort.

Admission National Institutes of Health Stroke Scale (NIHSS: > = 15 (poor) vs. <15 (good)), a commonly used scale to assess stroke severity, was significantly associated with higher overall cost in the subgroup (*p* < 0.001), and significantly associated with longer stay (unadjusted RR for longer stay: 1.41, 95% CI: 1.11 to 1.79), but was not associated with hypertension at admission (unadjusted RR for hypertension: 0.99, 95% CI: 0.78 to 1.24). Interestingly, hypertension was protective against in-hospital mortality in this subgroup (unadjusted RR for death: 0.23, 95% CI: 0.07 to 0.81) although there were only 9 patients who died in this group suggesting that these findings may be uninformative. However, it was noted that hypertension was also not associated with in-hospital mortality in the entire cohort (unadjusted RR for death: 0.90, 95% CI: 0.73 to 1.11).

Amongst those who survived in the subgroup (*n* = 139), Modified Rankin Scale (mRS: 3-5 (poor) vs 0-2 (good)), a commonly used measure of disability assessed at hospital discharge, was significantly associated with higher overall cost (*p* < 0.001) and longer length of stay (unadjusted RR for longer stay: 2.05, 95% CI: 1.46 to 2.88), although, like in-hospital mortality, was also not associated with hypertension (*p* = 0.256).

### Sensitivity analysis with all ICH admissions across 100 years

To address the issue of potentially below-satisfactory (<90%) statistical power for the primary exposure of interest, hypertension, possibly due to an insufficient sample size given the variability of cost data and effect size we observed, we used Monte Carlo methods to predict the total cost of hypertension adjusted for age in a hypothetical cohort of ICH patients over one generation (100 years) and compared this estimate to the estimate generated from our data.

To develop the cohort we assumed a theoretical random sample of 717,727 ICH patients over a 100-year period, which assumes a 0.94% population growth per year [13], and a hospitalization rate of 12 per 100,000 inhabitants per year within Canada which would be unchanged overall across the generation despite declines in some specific groups of patients [14, 15]. These estimates were assumed and entered into the simulation model based on previously published incidence rates within Canada [14], Canadian demographic data [13], as well as estimates derived from studies of large cohorts within North America [15]. For the purpose of the simulation, we assumed there was a real age-adjusted association between hypertension and total inflation-adjusted cost, but did not make an assumption as to the actual cost due to hypertension or its variability. The random sample of ICH patients were drawn from a normal population with an average age of 70 years, and standard deviation of 13 years [16]. All patients were between the ages of 18 and a theoretical human maximum of 126 years [17]. Hypertension occurred with an overall frequency of 77% in this group [15]. Like the primary analysis, all costs for the sensitivity analysis are reported in 2015 US Dollars for comparison.

One million unique samples of 717,727 ICH patients were drawn. Using these data, we estimated the average dollar increase per discharge for hypertension was $12,027.27; which was higher but not significantly different (*p* = 0.516; using a Welch’s mean comparison test assuming unequal variances) from our estimate derived from primary data; $10,324.56 (Table 3).

## Discussion

This study demonstrated that additional morbidities may be associated with longer stay and higher cost of hospital care overall after hemorrhagic stroke; with hypertension being the most frequent and costly multimorbidity. Patients with ICH arriving at hospital with hypertension were 31% more likely to stay in hospital beyond 1 week and cost an average of $8123.51 more per visit compared to non-hypertensive patients, when accounting for age and other morbidities. Most importantly, this cost increase was apparent at all stages of care from diagnosis to in-hospital rehabilitation. Although the data were collected for one time frame (one decade), analogous to a cross-sectional study design, we can assume the exposures (morbidity, length of stay, stroke severity, in-hospital mortality, disability) preceded the outcome (cost) in all cases; as patients cannot incur costs until they have been seen by a healthcare professional and received care.

As suggested in this study and others [18], it is reasonable to believe that the overall cost of ICH care increases as the total number of morbidities increases; since patients who are more sick would naturally require more care and additional resources. However, if the additive, equal-contribution assumption of multimorbidity were true, the adjusted cost increase for each multimorbidity would be the same across all multimorbidities. This study illustrates the contrary; suggesting that the adjusted cost to treat hypertension is different by $1486.38 on average per discharge compared to secondary cerebrovascular disease for example. Although this difference overall may not be statistically significant, owing to high cost variability, it may be clinically meaningful and exemplify differences in how these patients are cared for. Perhaps there are other treatment-process factors contributing to increasing costs in patients with specific multimorbidities beyond their overall number of additional diseases at admission which warrants further investigation.

We examined the risk of longer stay for different multimorbidities since length of stay in hospital is often cited as one of the most important predictors of treatment costs after ICH [19]; such that, those who stay longer in hospital may be significantly more costly to treat overall compared to those who stay for a shorter period of time. Often costs are assumed to fit normal parameters for these analyses; such that cost data are assumed to be normally distributed when compared to time spent in hospital. It would be inappropriate to compare raw costs to time if it were skewed as it was in our study, thus for this study all costs were transformed on the log scale prior to comparison to allow for a better examination of the relationship between cost and time.

When examining the association between length of stay and log cost we noted that these variables were not linearly related (Fig. 1). Further, the variability in cost appeared to be higher when hospital stay was short and lower when hospital stay was long, thus we did not assess this relationship using normal linear models. Previous authors have been less conservative and reported cost-per-day estimates overall for ICH and ischemic stroke patients [19–21]. These estimates assume cost data are normally distributed and linearly associated with time. Our data suggests that the greatest costs-per-day after ICH are likely incurred during the first day, conceivably due to high diagnostic resource use, and the magnitude of the increase in cost may decline as time progresses and is perhaps not significant after the third week of care; which is likely indicative of a curvilinear relationship. It should be noted that the significance of these findings is likely related to a reduction in sample size over time, such that, the probability of being discharged from hospital, and thus no longer contributing to cost, increases as time goes on; potentially affecting our ability to predict costs long after hospital admission.

Not surprisingly, in our exploratory analysis we illustrate that baseline stroke severity and disability was associated with overall cost and longer stay. One would expect that patients who are in serious condition when they arrive at hospital would likely be provided with the most aggressive, diverse, and potentially costly care overall compared to those with mild ICH. This analysis also suggested that stroke severity may not be acting as a confounder for the association between hypertension and cost since admission NIHSS was not associated with hypertension. Also, hypertension was not associated with in-hospital mortality or disability; suggesting that neither death nor disability could be acting as confounders in the hypertension-cost relationship. These exploratory analyses demonstrate a lack of clarity we have as to why costs are potentially higher for patients with stroke and hypertension. Despite this, it does shed light on an interesting health services phenomenon worth investigating in more detail in future studies. It is recommended the results from these analyses be used to generate discussion and guide future studies of this phenomena versus being interpreted as firm evidence to change practice as these observations, although interesting, are based on an exploratory analysis of a small subgroup of ICH patients in a single center without considering the impact of specific treatments.

Our study was limited in that we did not assess the association between clinical stroke severity or morbidity, and cost in the entire cohort as we only started recording this information in the latter half of the decade; and thus did not have complete data. Further, hypertension in this study was captured using ICD codes from administrative data and thus not defined using direct blood pressure measurements. Thus our definition of hypertension depended on the accurate diagnoses of responsible physicians. Further, as with all studies that use administrative data, we cannot rule out misclassification of diagnoses, and thus presume that patients classified as hypertensive are those presenting with hypertension and not those with only a history of hypertension. Although we attempted to identify patients with spontaneous ICH, we also cannot rule out the inclusion of some patients with other forms of primary intracranial hemorrhage due to the limitations of administrative data sources. As with nearly all centers, it is likely that many difficult to diagnose diseases, such as mood disorders, were under represented in our data which may affect the cost variability observed. Finally, we did not examine the impact of stroke physician fees on ICH costs as it was not possible to link patients with specific stroke physicians.

The primary cost data for this study was collected up to the year 2008 and inflated to the end of 2015. We believe our data is valid given the lack of meaningful changes in ICH care over the past several decades due to a lack of robust effective treatments available [4, 22, 23]. Despite this, we still took a conservative approach and assumed it could have been possible that patients may have changed over time, and thus informed our sensitivity analysis using current data collected at other centers. Using this approach, we found our data may in fact underestimate the impact of hypertension on cost; albeit not significantly.

## Conclusion

Patient multimorbidity, specifically hypertension, is a strong predictor of cost after spontaneous ICH. The greatest costs after ICH are incurred during the first week and the magnitude of the increase in cost declines overtime, thus time and cost are not linearly related. These factors should be considered when forecasting health spending for stroke.
